# Artificial Neural Networks Elucidated the Essential Role of Mineral Nutrients versus Vitamins and Plant Growth Regulators in Achieving Healthy Micropropagated Plants

**DOI:** 10.3390/plants11101284

**Published:** 2022-05-11

**Authors:** Tomás A. Arteta, Radhia Hameg, Mariana Landin, Pedro P. Gallego, M. Esther Barreal

**Affiliations:** 1 Agrobiotech for Health, Department of Plant Biology and Soil Science, Faculty of Biology, University of Vigo, 36310 Vigo, Spain; tarteta@uvigo.es (T.A.A.); hameg_radia87@yahoo.fr (R.H.); 2 CITACA—Agri-Food Research and Transfer Cluster, Campus da Auga, University of Vigo, 32004 Ourense, Spain; 3 Departamento de Farmacología, Farmacia y Tecnología Farmacéutica, I+D Farma (GI-1645), Faculty of Pharmacy, iMATUS and Health Research Institute of Santiago de Compostela (IDIS), Universidade de Santiago de Compostela, 15782 Santiago de Compostela, Spain; m.landin@usc.es

**Keywords:** artificial intelligence, basal medium composition, in vitro culture medium, mineral nutrition, modeling, neurofuzzy logic, plant tissue culture

## Abstract

The design of an adequate culture medium is an essential step in the micropropagation process of plant species. Adjustment and balance of medium components involve the interaction of several factors, such as mineral nutrients, vitamins, and plant growth regulators (PGRs). This work aimed to shed light on the role of these three components on the plant growth and quality of micropropagated woody plants, using *Actinidia arguta* as a plant model. Two experiments using a five-dimensional experimental design space were defined using the Design of Experiments (DoE) method, to study the effect of five mineral factors (NH_4_NO_3_, KNO_3_, Mesos, Micros, and Iron) and five vitamins (Myo-inositol, thiamine, nicotinic acid, pyridoxine, and vitamin E). A third experiment, using 20 combinations of two PGRs: BAP (6-benzylaminopurine) and GA_3_ (gibberellic acid) was performed. Artificial Neural Networks (ANNs) algorithms were used to build models with the whole database to determine the effect of those components on several growth and quality parameters. Neurofuzzy logic allowed us to decipher and generate new knowledge on the hierarchy of some minerals as essential components of the culture media over vitamins and PRGs, suggesting rules about how MS basal media formulation could be modified to assess the quality of micropropagated woody plants.

## 1. Introduction

*Actinidia arguta* (Sieb. et Zucc.) Planch. ex Miq, known as the hardy kiwi, is a deciduous climbing plant native to China, Japan, Korea, and Siberia [1]. The fruit is small and hairless, thus it can be eaten as a whole without peeling [2,3]. Although recent studies have focused on the micropropagation of this species, an optimized culture medium for its multiplication is not yet formulated.

The development of a suitable culture medium for plant tissue culture implies the combined use of multiple factors such as mineral nutrients, vitamins, or plant growth regulators. These components interact in a complex and often in a hidden way [4]. The optimization of basal media has been a difficult task since its beginning around 1900. Deciphering the role of each component of the culture medium would lay the foundations for the design of suitable media to obtain healthy micropropagated plants [5,6].

Due to a large number of variables involved in the development of such complex media, some computer-based tools such as response surface methodology [7,8,9] or Chi-squared automatic interaction [10,11] have been introduced for plant tissue researchers to decipher the importance of media components on the growth and quality of tissue-cultured plants, avoiding the limitations of traditional statistics and response surface methodologies [12,13]. Recently, some machine learning tools based on artificial intelligence (AI) algorithms open new horizons to the plant biotechnology field since they seem to be able of solving the problems that arise during the development of a new culture medium, achieving smart solutions for new species or cultivars [14]. Artificial Neural Networks (ANNs) have certain advantages over other approaches [15]. These tools are flexible and versatile, allowing new results to be incorporated into the previous database and re-analyzed to extract additional information, creating new and useful knowledge [16].

For example, Gago et al. [17] were able of identifying the key factors for simultaneous rhizogenesis and acclimatization of *Vitis vinifera* using neurofuzzy logic technology, which combines artificial neural networks and fuzzy logic algorithms. ANN tools combined with the data mining strategy also allowed them to evaluate the effect of culture media composition on plant growth parameters of various apricot cultivars [18]. 

Later, combining DoE and neurofuzzy logic technology, Nezami-Alanagh et al. [19] were able to establish the specific effect of each ion of culture media on shoot multiplication of *Pistacia vera*, but also on the appearance of physiological disorders of pistachio rootstocks cultured in vitro.

Recently, in a previous study carried out in our lab, Hameg et al. [4] successfully applied this methodology to study the mineral nutrition of *A. arguta*, proving that the newly developed R medium for this species, which differed from MS basal medium [20] by reducing the nitrogen content and increasing Mesos and Iron concentration, performed better for kiwiberry micropropagation.

In addition to mineral nutrients, whose effects on different plant species have been widely studied, vitamins constitute an essential component of most plant tissue culture media [5]. The type or amount required for the plant remains unclear [21]. In a recent study, Arteta and coworkers [22], taking advantage of ANN tools, shed light on the role of certain vitamins such as pyridoxine, vitamin E, and Myo-inositol on the shoot number and shoot length of *A. arguta*.

Plant Growth Regulators (PGRs) are vital organic compounds synthesized by plants, which play an essential role in their differentiation and development at low concentrations [23,24]. The addition of suitable PGRs to the culture media has been effective in regenerating kiwiberry shoots [25]. It is widely accepted that their addition is required for successful shoot initiation and subsequent proliferation [26,27]. Here, the effect of two PGRs, the cytokinin 6-benzylaminopurine (BAP) and the gibberellin gibberellic acid (GA_3_), was studied to elucidate their importance on healthy kiwiberry micropropagation.

In this study, it has been hypothesized that although MS medium performs reasonably well, its composition (mineral, vitamins, and PRGs) could be modified to improve the quality of micropropagated plants, avoiding the morphophysiological disorders described in some woody species [19,28], also in arguta [4], when MS was selected as basal culture medium. For this, a strategy based on data mining was applied. Data from two previous studies focused on the effect of mineral nutrients [4] and vitamins [22] on the micropropagation of *A. arguta* were merged with the results of a new experiment focused on the effect of two PGRs. All treatments were established based on the original MS formulation. A new and unique database was generated and modeled using a neurofuzzy logic tool to better understand the role and importance of mineral nutrients, vitamins, and PGRs. Neurofuzzy logic could decipher the critical variables that determine the healthy growth of micropropagated plants, generating rules on whether or not to modify the original formulation of MS medium. The computer-based tool (ANNs) that have been used to study how MS basal media formulation could be modified to assess the quality of micropropagated woody plants.

## 2. Results

**Figure 1:**
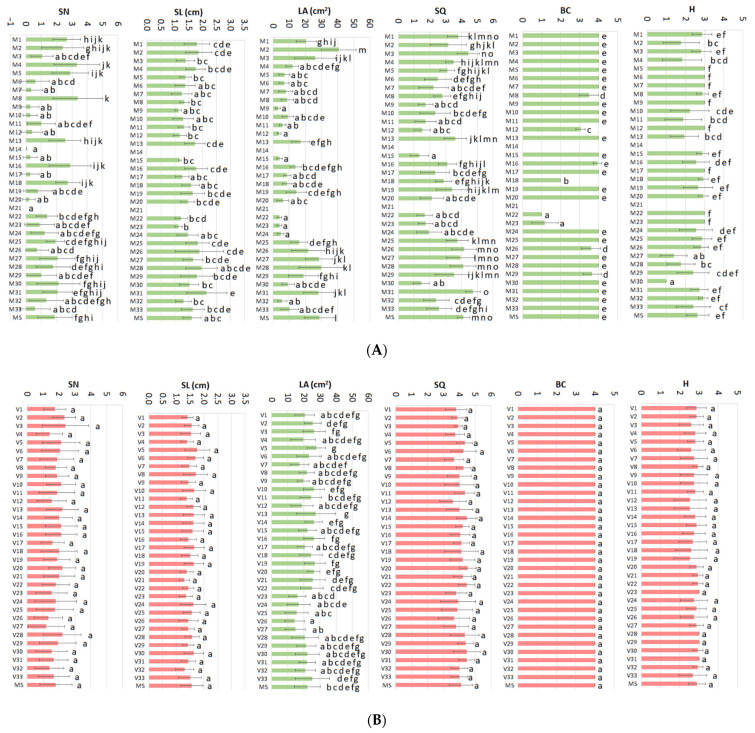

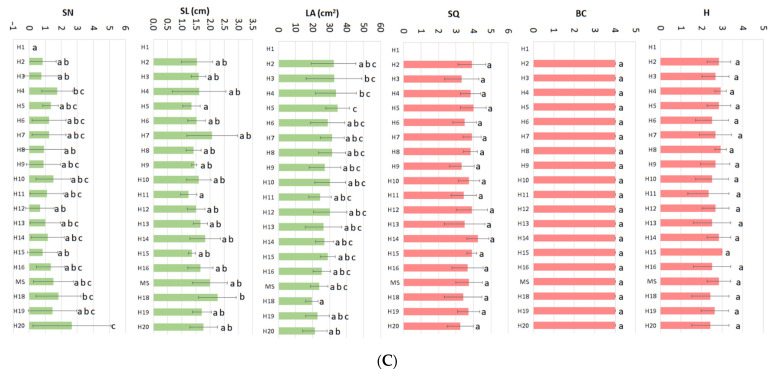
Average results for the different treatments of the mineral nutrients database (**A**), the vitamins database (**B**), and the PGRs database (**C**) for all parameters measured (*SN: shoot length, SL: shoot length, LA: leaf area, SQ: shoot quality, BC: basal callus, H: hyperhydricity*). Green graphs indicate statistically significant differences among the treatments, red graphs are the opposite. Different letters indicate statistically significant differences (*p* < 0.05).

Results of fractional statistical analysis (ANOVA) revealed that while mineral nutrient variations caused statistically significant effects on all the parameters studied (Figure 1A, green color), vitamins caused effects only on the leaf area parameter (Figure 1B, green color). PRGs caused significant effects on the growth parameters (Figure 1C, green color).

In this study, despite having taken special care to select the most homogeneous material possible in terms of explant size as well as in the determination of the response parameters (see data acquisition in the Section 4), it has been evident a great variation in the values determined for each one of the parameters. This great variance difficult to describe which treatment caused the best response (e.g., Figure 1A, SN). Several treatments produced better results than MS for several parameters (for example, M31, Figure 1A), but not for all. This makes it very difficult for a researcher to select the combination of components that would produce the best response for each parameter to design a formulation better than MS. The interpretation of the results of the statistical analysis has been difficult and has made it impossible to identify the critical variables or establish the optimal combination of mineral nutrients, vitamins, and PGRs for the healthy micropropagation of *Actinidia arguta*.

Neurofuzzy logic succeeded in modeling the six growth and quality parameters of *A. arguta* as a function of the mineral ions, vitamins, and PGRs concentrations (Table 1). Model Train Set R^2^ values were higher than 70%, considered a high model predictability indication [18]. Furthermore, all calculated *f*-ratios were higher than the *f* critical values (α = 0.01), confirming the model quality and accuracy as there are no statistically significant differences between predicted and experimental values.

Differences in growth parameters are mainly explained by variations in mineral nutrients and PGRs and also in some vitamin concentrations, being pointed out as critical factors by neurofuzzy logic. For the SN, the model achieved a high Train Set R^2^ (82.3%) and generated four submodels being the interaction between Fe^2+^ and Na^+^ the one with the highest contribution. The model established other additional submodels, with lower contributions: the interaction between K^+^ and SO_4_^2−^ and the independent effect of GA_3_ and BAP (Table 1).

Eight different submodels were generated for the SL parameter (R^2^ = 70.3%), being the Co^2+^ the variable with the highest effect on this parameter. Other submodels established by the model were the ones showing the independent effect of Na^+^, Mg^2+^, BO_3_^−^, Vitamin E, and Myo-inositol, and two submodels showing the interaction between NO_3_^−^ and K^+^ and between GA_3_ and BAP, respectively (Table 1).

For the LA, the interaction between K^+^ and NO_3_^−^ was the main factor (R^2^ = 77.7%). Besides, it was also established the independent effect of Na^+^, SO_4_^2−^, and GA_3_ on the LA, but their contribution was lower (Table 1).

Neurofuzzy logic excluded vitamins as critical factors for the morphophysiological quality responses, including only minerals and PGRs. For the SQ (R^2^ = 85.6%), six submodels were generated, being the effect of NO_3_^−^ the one with the highest contribution. Five additional submodels included the independent effects of four ions: K^+^, NH_4_^+^, Fe^2+^, MoO_4_^2−^, and one PGRs: BAP.

For the BC (R^2^ = 96.0%), neurofuzzy logic generated two submodels, the interaction between PO_4_^3−^ and NH_4_^+^ as the one with the stronger contribution, and the independent effect of SO_4_^2−^.

The hyperhydricity model included five submodels (R^2^ = 84.4%), being the interaction SO_4_^2−^ and NO_3_^−^ the main factor. The four additional submodels involved the interaction between Co^2+^ and NH_4_^+^, between Ca^2+^ and Fe^2+^, and the independent effect of I^−^ and BAP (Table 1).

Together with the Train Set R^2^, ANOVA parameters and the selection of the critical factors, FormRules^®^ software generates simple ‘IF THEN’ rules which described how the critical factors (ions, vitamins, PGRs, and their interactions) affect each output. Rules are shown in Table 2 and Table 3.

As it was mentioned, the SN parameter was mainly explained by the interaction between Fe^2+^ and Na^+^ (Table 1). The model showed the positive effect of Low Na^+^ on the shoot regeneration when combined with any level of Fe^2+^ tested, except for the combination of High Na^+^ with High Fe^2+^, which also promotes the shoot formation (Table 2, rules 1, 3, 5, and 6). The meaning of High, Mid, and Low terms can be consulted in Table S1, in which the limit values of each one has been included as Supplementary Information for a better understanding. The model also highlighted the independent and positive effect of High GA_3_ on SN parameter (Table 2, rules 9), and the negative effect of BAP at any concentration (Table 2, rules 19 and 20). Finally, an inverse relationship between K^+^ and SO_4_^2−^ has been pointed out. To favor new shoot proliferation Low, Mid, and High levels of K^+^ should be combined with High, Mid, and Low levels of SO_4_^2−^ respectively (Table 2, rules 12, 14, and 16), while any other combination leads to lower shoot proliferation (Table 2, rules 10, 11, 13, 15, 17, and 18).

SL is also highly dependent on the Co^2+^ concentration in the media. Low concentrations should be used to achieve the highest SL (Table 2, rule 43). The model also stated the independent effect of three ions: the positive effect of Mid-High BO_3_^−^ and High Mg^2+^ (Table 2, rules 25, 33, and 34) on the SL, and Low-Mid concentrations of Na^+^ (Table 2, rules 21 and 22). The neurofuzzy logic model established as positive to obtain long shoots, the inverse relationship between NO_3_^−^ and K^+^. In order to obtain longer shoots, Low-High K^+^ should be combined with High-Low NO_3_^−^ (Table 2, rules 27 and 28). Other ratios worsen shoot sizes. The interaction between PGRs has also an important effect on shoot size (Table 2, rules 35, 40, and 42, respectively), being some of the following combinations necessary to promote a High SL:

(i) Low BAP and Low GA_3_
(ii) Mid_2 BAP and High GA_3_
(iii) High BAP and High GA_3_

Finally, when the media was supplemented with Low Vitamin E and Myo-inositol, High SL was promoted (Table 2, rules 30 and 46).

The leaf area parameter is affected negatively by Na^+^ ion concentration. Low Na^+^ concentrations are recommended to achieve High leaf area (Table 2, rule 48). The neurofuzzy logic established that a High concentration of NO_3_^−^ in combination with any level of K^+^ (Table 2, rules 53 and 55) and the independent effect of High-level of SO_4_^2−^ (Table 2, rule 57), were necessary for obtaining a High LA. Eventually, the rules described a negative effect of the GA_3_ on this parameter, showing that Low levels of GA_3_ promoted the largest LA (Table 2, rule 50).

The predictability of the models of morpho-physiological responses is even higher than those of the growth parameters as can be assessed by the Train Set R^2^ values (Table 1). NO^3−^ ion concentration has been selected as the most critical factor affecting the shoot quality, being necessary to maintain Low to Mid concentrations of this ion to achieve the High SQ parameter (Table 3, rules 10 and 11). Other submodels stated the independent effect of four ions (NH_4_^+^, K^+^, MoO_4_^2−^, and Fe^2+^) and one PGR (BAP). The rules established that to achieve high-quality shoots it was necessary to supplement the media with Low Fe^2+^, High K^+^ and NH_4_^+^, Mid MoO_4_^2−^, and Low BAP (Table 3, rules 1, 4, 6, 8, and 13).

**Figure 2:**
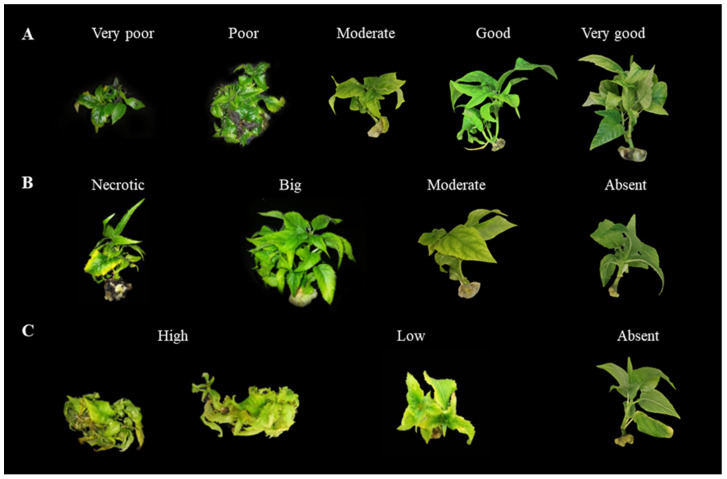
Shoot quality rating (**A**): 1(very poor). 2 (poor). 3 (moderate). 4 (good) and 5 (very good); basal callus formation rating (**B**): 1 necrotic). 2 (big). 3 (moderate) and 4 (absent) and hyperhydricity rating (**C**): 1 (high). 2 (low) and 3 (absent).

Basal callus formation and hyperhydricity are two parameters that evaluate the appearance of physiological disorders and were included to estimate the negative effect of some medium components on the final quality of the micropropagated plantlets (Figure 1). To facilitate reader understanding, High BC (up to 4) or H values (up to 3) mean plantlets of excellent quality. On the contrary, low values (0) mean poor quality due to the appearance of necrotic basal callus and/or high hyperhydricity symptoms (Figure 2).

The interaction between NH_4_^+^ and PO_4_^3^ has the strongest effect on the BC parameter, being the combination of Low NH_4_^+^ and Mid_3-High PO_4_^3−^ the best one to avoid the presence of basal callus (Table 3, rules 21–26). The model pinpointed that SO_4_^2−^ was necessary for achieving healthy plantlets (Table 3, rules 27–29).

The neurofuzzy logic model determined an interaction between SO_4_^2−^ and NO_3_^−^ on hyperhydricity. The disorder can be avoided (High H) maintaining a High SO_4_^2−^ ion concentration in the medium, independently of the concentration of NO_3_^−^ (Table 3, rules 36 and 37). The model stated that hyperhydricity was also avoided by the interaction of Low Co^2+^ with any concentration of NH_4_^+^ (Table 3, rules 48 and 49), as well as the interaction between Low-Mid_2 Ca^2+^ and any level of Fe^2+^ (Table 3, rules 38–41). High I^−^ also caused a positive effect on this parameter (Table 3, rule 31). Finally, Low BAP caused low to no hyperhydricity (Table 3, Rules 46).

## 3. Discussion

Murashige and Skoog (MS) [20] is a very well-designed medium for plant tissue culture, being cited in over 88.000 publications according to Google Scholar web search engine. Nonetheless, it seems to be unsuitable for some species, due to the occurrence of physiological disorders such as shoot tip necrosis or hyperhydricity [27,28], and for being supra optimal for some kiwifruit species [29,30]. Some authors have reported that it is necessary to reduce its composition by half or even more to enhance plant micropropagation [31,32,33]. A wide range of strategies has been implemented to improve plant tissue culture protocols by modifying the composition of the most commonly used basal media, such as One-Factor-At-a-Time (OFAT) [34]. However, this strategy of studying a single or a few factors has several drawbacks, since it only provides reduced information on the partial “optimum” of each factor, ignoring the interactions between them and increasing exponentially the number of treatments to be evaluated [35]. Over time, this strategy was almost abandoned because plant basal media design requires a multivariate approach, as has been demonstrated [12,13].

The use of DoE to modify and improve the MS culture medium reduces the number of treatments but, at the same time, assesses an adequate sampling of the design space [36,37]. Recently, this methodology was applied successfully in our lab [4], to design an optimized R medium and to improve the mineral nutrition of *Actinidia arguta*. The mineral content of this medium reduced by 20% the nitrogen content but increased by 200% the Mesos (CaCl_2_·2H_2_O, MgSO_4_·7H_2_O, KH_2_PO_4_), by 100% the Micros (MnSO_4_·4H_2_O, ZnSO_4_·7H_2_O, H_3_BO_3_, KI, CuSO_4_·5H_2_O, Na_2_MoO_4_·2H_2_O, CoCl_2_·6H_2_O) and by 50% the Iron (FeSO_4_·7H_2_O, Na_2_·EDTA) compared to MS. However, the variation of other medium components such as vitamins and PGRs, which might modulate the effect of the mineral nutrients, were not included in that database.

In this study, it has been hypothesized that although MS medium performs quite well, its composition (mineral, vitamins, and PRGs) could be modified to improve the quality of the micropropagated woody plantlets, avoiding the morpho-physiological disorders described in some woody species [16], and also in arguta [4], when MS was used as culture medium. To that end, a strategy based on data mining was used. Data from two previous studies focused on the effect of mineral nutrients [4] and vitamins [22] on the micropropagation of *Actinidia arguta* were merged with the results of a new experiment focused on the effect of two PGRs. It should be noted that some modifications have been made compared to previous databases: (i) EDTA has been removed as a factor and only Fe^2+^ ion is considered, (ii) shoot number (SN) and shoot length (SL) parameters have been curated to better represent the most viable shoots for subsequent stages of micropropagation (see Material and Methods). All treatments were established based on the original MS formulation. A new and unique database was generated, which was modeled using a neurofuzzy logic tool to decipher the critical variables (mineral nutrient, vitamin, and PGR) that determined the healthy growth of micropropagated woody plants and to obtain some rules on whether or not to modify the original formulation of MS medium and how to do it.

The statistical analysis carried out through ANOVAs shows that there are statistically significant differences between treatments for the growth and quality parameters of the micropropagated plants (Figure 1). Particularly, the variations in the mineral nutrients seem to have significant effects on the whole set of variables, followed by the PGRs (3 out of six) and the vitamins (only 1 out of 6). ANOVA does not allow easy interpretation of the results, since it indicates which treatments lead to the same or different results, but not which factors cause the detected effect. Thus, by using this traditional ANOVA strategy is practically impossible to select the best overall treatment which fulfills all the requirements for all studied parameters, as demonstrated here.

Artificial neural network tools such as neurofuzzy logic emerged as a novel strategy able to manage big databases and find hidden trends between variables, pointing out the importance of certain medium components [16,28]. Thus, each treatment was split up into a set of factors that include the concentration of each component. Twenty-four factors, of which 17 are mineral ions, 5 are vitamins and 2 are PGRs were used as inputs to model growth and quality parameters. Accurate models allow the selection of the critical factors and complement the statistical analysis. The structure of the global experimental design (3 independent experiments) does not allow establishing the effect of interactions between mineral nutrients, vitamins, and PGRs, but it does reveal a hierarchy regarding the importance of a particular component or group of components.

The set of critical factors selected by the neurofuzzy logic models (Table 1) includes 13 out of 17 mineral nutrients (excluding Cl^−^, Cu^2+^, Mn^2+^, and Zn^2+^ as key factors), 2 out of 5 vitamins, and the two PGRs. Among components explored, nitrogen sources (NO_3_^−^ and NH_4_^+^) seem to have special importance as they were included in 5 out of 6 parameters, followed by SO_4_^2−^, K^+^, and BAP in 4 out of 6. Fe^2+^, Na^+^, and GA_3_ affected 3 out of 6 parameters, while Co^2+^ only affected 2 out of 6. Other medium components (Ca^2+^, PO_4_^3−^, Mg^2+^, BO_3_^−^, MoO_4_^2−^, I^−^, Myo-inositol, and vitamin E) are involved in just 1 out of 6 parameters. The main role of mineral nutrients, over vitamins and PGRs, was demonstrated.

Nitrate, ammonium, potassium, and sulfate ion and the interactions between them affected all parameters studied, so the model reveals their importance in agreement with previous in-house results [4].

Nitrogen sources (NO_3_^−^ and NH_4_^+^) are constituents of proteins, nucleic acids, and chlorophyll, being crucial to plant life [5]. Neurofuzzy logic established that NO_3_^−^ affected both growth and quality parameters (SL, LA, SQ, and H). The importance of this ion has been recently reported by several authors. For pistachio rootstocks, Nezami and collaborators [28] determined that levels of NO_3_^−^ around 35 mM, in combination with 0–0.3 mM Fe^2+^ and Cu^2+^ ranged from 0.1–0.3 µM, were needed to improve shoot length. Here, the optimal ranges for *A. arguta* suggest that it could be maintained up to the MS levels (39.41 mM; Table 4), without interacting neither with Fe^2+^ and Cu^2+^. The differences in the interactions shown by the model compared to pistachio are probably due to the limitation of the number of factor interactions in the model training parameters (3 versus 2 in the present study), or the possible different nutritional requirements of these two different woody species. Silvestri et al. [38] did not find significant differences in shoot length with variations in NH_4_NO_3_ and KNO_3_, in in vitro micropropagation of *Corylus avellana*. This lack of significant results might be due to the use of elevated KNO_3_ salt concentration in that study, well above the ranges used in the present study, which may lead to the conclusion that concentrations above KNO_3_ MS levels do not affect the shoot length.

Interestingly, the nitrate ion did not interact with the other nitrogen source in the in vitro culture media, the ammonium ion (NH_4_^+^), although they share one mineral salt (NH_4_NO_3_). Contrary to NO_3_^−^, ammonium ion only affected morphophysiological parameters. The model established that NH_4_^+^ interacts with PO_4_^3−^ affecting the basal callus (BC) and with Co^2+^ affecting the hyperhydricity (H). The variability of these two parameters was entirely explained by phosphate and cobalt ion, independently of NH_4_^+^ levels. Although cobalt is not considered an essential element in plant tissue culture, is a component of vitamin B12 which is involved with nucleic acid synthesis [39]. Evidence of its stimulatory effect on the growth and differentiation of plant tissue cultures is hard to find [5]. In this study, Co^2+^ levels over 0.08 µM (Table 4 and Table S1) induced shoot hyperhydricity.

Another abnormality involving NH_4_^+^ ion was the induction of BC. The presence of this ion interacting with PO_4_^3−^ above 1.60 and up to 3.75 mM (Table 4 and Table S1) concentrations reduced the basal callus. Our previous studies working with ions corroborate the use of 1.17–3.75 mM PO_4_^3−^ to avoid big/necrotic callus [4]. Other authors reported that basal callus was stimulated by 5× levels of MS KH_2_PO_4_ (6.25 mM), although the tested levels in that study exceeded the 3× assayed in the present work [40], which probably proves that the optimal range is restricted to 12.37–20.61 mM.

Another nitrate ion interaction was the one involving K^+^. It is worth noting that the interaction between NO_3_^−^ and K^+^ was critical for two different parameters: SL and LA, and they both independently affected the SQ. Potassium has been described as an essential factor controlling plant growth [41]. Potassium and nitrate ions share the same salt, potassium nitrate (KNO_3_), although each one of them is present in other media salts (NH_4_NO_3_, KH_2_PO_4_, KI). The role of some of these salts has been widely discussed in different studies, using a large variety of plant species such as stevia [42], pear [7], and barley [40], but a clear comprehension and understanding of their effect have not been retrieved. This could be due to those reports discussing the results based on the effect of the salt, rather than the effect of the individual ions that form the salts. It is obvious, that any change in the concentration of one of the salts will always affect, in this case, at least the two ions that constitute it, but also the total concentration of that ion in the medium. Over the years, it has been almost impossible to make decisions or establish precise and accurate cause-effect relationships on the role of mineral nutrients since most studies are based on the salt composition of the medium... This phenomenon is known as ion confounding [36], and it can be avoided by working with ion data instead of salt data. Recently, various studies began to discern the specific effect of individual ions. Akin and collaborators [10] reported that hazelnut plant shoot quality improved when K^+^, NH_4_^+^, and NO_3_^−^ ions were added at precise concentrations (K^+^ ≤ 46 mM, NH_4_^+^ ≤ 20 mM, and NO_3_^−^ ≤ 88 mM) to the culture media. These results disagree with the optimal ranges for these ions in the present study (Table 4), and also with the previous ones [4], demonstrating that ions are more useful to identify cause-effect relationships rather than salts.

Some previous studies pointed out the beneficial effect of increasing the concentration of Meso salts of MS medium (MgSO_4_, CaCl_2_, KH_2_PO_4_) to improve the number of shoots [43]. Hunková et al. [44] indicated the superiority of using a treatment of MSx3 Mesos components (MgSO_4_, CaCl_2_, KH_2_PO_4_) versus MSx4 on the in vitro growth of several berry fruits, and the greater number of shoots that gives rise to for *Amelanchier alnifolia*. But here, NO_3_^−^ also interacted with SO_4_^2−^, affecting the hyperhydricity, and the latter also interacted with K^+^, affecting the SN. To the best of our knowledge, these effects never have been reported.

The sulfate ion is also known to have a positive effect on callus formation in different species [43,45,46]. Previous studies proved that the presence of SO_4_^2−^ (0.49–5.20 mM) reduced the formation of basal callus for *A. arguta* [4], an effect also described in the present study, although it should be at 2.85–5.20 mM (Table 4) to achieve the best results for the rest of the parameters. It is worth noting that the model training parameters were adjusted from 4 maximum inputs per submodel in that study [4] to just 2 in this study (see training parameters in the Section 4) This model adjustment was done to simplify the rules and to clarify which minerals are crucial. The implications of this adjustment can be observed in the effect of K^+^ over SQ. Although in our previous study, the positive effect of K^+^ on interaction with SO_4_^2−^ was pointed out for SQ [4,22], in the present study sulfate ion did not appear as a key factor affecting this parameter, probably underlining the predominant role of K^+^, as this ion persisted as critical for this parameter in both studies. In the present study, a strong interaction of K^+^ with both NO_3_^−^ and SO_4_^2−^ was described, being necessary to have Low K^+^ levels and High NO_3_^−^ and SO_4_^2−^ or *vice versa*, to achieve the highest results for SN, SL, and LA. For SQ, High levels of K^+^ always should be supplemented. Overall, K^+^ supplemented at Mid-range (7.28–17.46) mM is highly recommended (Table 4 and Table S1).

As discussed above, the importance of Mesos was demonstrated in several studies [43,45,46], but since the authors based their conclusions on salts, the ion confounding effect arises and no clues about the effect of single ions can be achieved, such as Mg^2+^. Magnesium is an essential component of plants as part of the chlorophyll molecule and is crucial for the activity of many enzymes and necessary for maintaining the integrity of ribosomes [5]. Neurofuzzy logic established the importance of this ion in the culture medium, being necessary to supply Mg^2+^ at 2.44–4.50 mM (Table 4) to achieve longer shoots. That optimal range is slightly higher than the one obtained for the same species in our previous studies [4,22]. This correction of the optimal range could be because the model now considers all the components of the medium (minerals, vitamins, and PGRs). Hidden interactions between all these components could determine the need for this small adjustment in magnesium concentration and suggest that the levels of Mg^2+^ can be infra-optimal in MS.

Micros such as Co^2+^ (discussed above), I^−^, MoO_4_^2−^, and BO_3_^−^ must be carefully adjusted for proper plant tissue culture because they are completely necessary but their optimal concentration range is narrow and minor variations can cause either toxicity or deficiency [47,48]. ANN tools identified the importance of these ions and established the optimal concentration ranges for successful shoot development. In this way range of 0.05–0.15 mM BO_3_^−^ (rule 33, 34, Table 2), 0.5–1.2 µM MoO_4_^2−^ (rule 8, Table 3), and 4.0–7.5 µM I^−^ (rule 31, Table 3 and Table S1), should be taking into account for plant micropropagation.

The neurofuzzy logic model established the interaction between Na^+^ and Fe^2+^ as the main submodel affecting the SN (Table 2, rules 1–6). Equimolar supplementation of the Fe^2+^ and EDTA components in the culture medium is mandatory to avoid iron precipitation [5,49]. Since only Fe^2+^ plays a physiological role in plant growth, only this ion was included in the database (Table S3). Variations in iron levels have been studied for different species with disparate results. Kothari and collaborators [50] concluded that shoot regeneration of *Eleusine coracana* L. was enhanced by quadrupling the Fe/EDTA MS levels. For other species such as red raspberries and *Gerbera hybrida,* an Fe/EDTA concentration higher than 1 mM was toxic, probably due to the EDTA, showing that MS levels (0.1 mM) were adequate to obtain high shoot number, length, and good quality [5,43,51]. Neurofuzzy logic established that 0.1–0.3 mM Fe^2+^ improved the shoot quality and stated the crucial effect of iron on the shoot number, but it is highly dependent on the interaction between other ions. The adjustment of iron concentration is a complex task, due to the known toxicity of EDTA and sodium, being this toxicity dependent on the species [28,52]. Some authors pointed out that the basal medium MS includes Na_2·_EDTA in excess (37.3 mg L^−1^) to chelate FeSO_4_·7H_2_O (27.8 mg L^−1^) [51]. MS medium (pH 5.8) seems to induce Fe^2+^ precipitation (up to 45%) due to at that pH the Fe/EDTA is not stable [47]. Recent studies have been conducted in which Fe/EDTA has been replaced by other chelators, such as Fe/EDDHA [53,54], which may be a compromise solution to facilitate the adjustment of iron salts in the in vitro culture medium, avoiding the toxic effect of EDTA at high concentrations.

Although most of the key factors were the mineral nutrients, PGRs also contribute to explaining the variability of five out of six parameters. According to the literature, gibberellins and cytokinin exert antagonistic effects on numerous developmental processes, including shoot and root elongation, cell differentiation, shoot regeneration in culture, and meristem activity [55,56]. But, although PGRs play an important role in shoot regeneration and elongation, their effect can be inhibited as a consequence of an imbalance in nutrient concentration [50,57,58]. This could explain why the neurofuzzy logic model not only stated BAP as detrimental for shoot multiplication (SN), despite being a cytokinin but also established that BAP at 0.50–1.50 mg L^−1^ caused shoot hyperhydricity. Several authors have suggested that cytokinins such as BAP might promote this phenomenon in plant tissue culture [59,60]. This study also supports that some physiological disorders, such as hyperhydricity, can be induced during plant micropropagation depending on the BAP levels in the medium.

Vitamins remain the least studied components of plant tissue culture medium and their role is currently unclear [21]. Our recent studies [22,61,62], carried out to assess the role of mineral nutrients and vitamins, provided new findings pointing out the positive effect of these organic compounds on the shoot number and length of *A. arguta*. ANOVA results show that variations in the vitamins within the limits of the study only significantly affect the leaf area of *A. arguta*. The ranges of Myo-inositol and vitamin E concentrations established by that ANNs model were readjusted with the new information provided by the PGRs data included in this database, suggesting that to achieve longer shoots, the media should be supplemented with up to 500 mg L^−1^ Myo-inositol and up to 0.5 mg L^−1^ vitamin E. It should also be noted that the model did not establish any interaction between PGRs and vitamins, as the experimental design was not conceived to that end. A much clearer cause-effect of vitamins and their interaction with other components of the medium could be achieved by developing a future single experimental design that includes all factors simultaneously (minerals, vitamins, and PGRs).

## 4. Materials and Methods

### 4.1. Plant Material and Stock Condition

Shoots of *Actinidia arguta* (Sieb. et Zucc.) Planch. ex Miq cv. Issai were micropropagated on Cheng stock medium [63], supplemented with 1 mg L^−1^ 6-benzylaminopurine (BAP) and 1 mg L^−1^ gibberellic acid (GA_3_), 8 g L^−1^ agar, and 30 g L^−1^ sucrose. The pH was adjusted to 5.8 before autoclaving (121 CC for 15 min at 105 KPa). The explants were cultured in 200 mL glass vessels containing 30 mL of medium each. The cultures were kept at 25 ± 1 °C under a 16 h photoperiod with 40 µmol m^−2^ s^−1^ irradiance provided by cool white fluorescent tubes, as previously described in detail [4].

### 4.2. Micropropagation Culture Conditions

Nodal segments of about 2 cm were cultured in 200 mL culture vessels containing 30 mL of each medium for 50 days. All treatments from all three experiments were supplemented with 2 mg L^−1^ glycine, 30 g L^−1^ sucrose, and 8 g L^−1^ agar. Control treatments were supplied with MS mineral nutrients and vitamins and with 1 mg L^−1^ BAP, and 1 mg L^−1^ GA_3_. The cultures were maintained at the same temperature and photoperiod as described above. 

Each treatment included five replicates of three explants each contained in glass vessels sealed with plastic caps. The experiments were carried out in triplicate. The shoots were harvested after 50 days.

### 4.3. Experimental Design and Data Acquisition

In this study we have combined in a new and unique database the results of three independent experiments carried out in our lab:

The first experimental design focused on the study of mineral nutrition [4]. Salts of MS medium [20] were classified into 5 independent factors (single salt or group of salts) as described elsewhere [4]: (i) NH_4_NO_3_, (ii) KNO_3_, (iii) Mesos, (iv) Micros, and (v) iron. Each factor had several levels corresponding to different concentrations of the MS medium (Table 5), following a D-optimal design [37] established through the software Design-Expert^®^ [64]. The generated database included 34 treatments, 33 generated by the software using a modified D-optimal design [7] plus 3 additional points of MS media used as controls (Table S2). The MS treatment data was calculated as the average of the three additional points. All treatments were supplemented with MS vitamins [20] and 1 mg L^−1^ BAP, and 1 mg L^−1^ GA_3_.

The second experimental design focused on the effect of vitamins [22]. The same design was used as in the previous case (D-optimal for 5 factors). In this case, the 5 independent factors were: Myo-inositol (Myo), thiamine (Thia), nicotinic acid (Nic), and pyridoxine (Pyr) plus a fifth one the vitamin E (Vit E) not present in MS medium (Table 5). As previously described, a database included 34 treatments (33 generated by the software plus 1 additional point (average of 3 treatments) of MS media used as control (Table S2). 

A third experiment was carried out to evaluate the effect of PGRs. The experimental space was designed to decipher the effect of extreme concentrations from very low (0 mg L^−1^) up to very high (2.5 mg L^−1^ BAP or 1 mg L^−1^ of GA_3_) on shoot growth and quality responses. Thus, 20 combinations of both PRGs were tested (Table S2).

Finally, mineral nutrient, vitamin, and PGR databases were merged into one single database, which ultimately contains the three different experimental designs mentioned (Tables S3–S5). This circumstance will prevent the model to detect any nutrient-vitamin, vitamin-PGR, or nutrient-PGR interactions, but as stated before, it should allow the selection of crucial components for the *A. arguta* healthy in vitro growth.

The following growth responses were evaluated as described previously [4] (Figure 2):

1. Shoots number (SN), number of new regenerated shoots per explant, longer than 1 cm.
2. Shoot length (SL), length from the base to the tip of the new regenerated shoots longer than 1 cm.
3. Leaf area (LA), the sum of areas of the leaves >1.5 cm was measured (cm^2^) for all the explants (the original and the new ones), using a portable laser leaf area meter (Meter CI-202, CID biosciences, WA, USA).

As the MS mineral salts have been reported for promoting physiological disorders in some plants [28], the next three morphophysiological quality responses were also evaluated in all the explants (the original and the new ones; Figure 2):

1. Shoot quality (SQ) as indicative of shoot vigor, was visually assessed, and scored from 1 to 5 (1 very poor, 2 poor, 3 moderate, 4 good, and 5 very good).
2. Basal callus (BC), callus formation at the cut edge of shoots was visually assessed and scored from 1 to 4 (1 necrotic, 2 big, 3 moderate, and 4 absent).
3. Hyperhydricity (H), was visually assessed and scored from 1 to 3 (1 high, 2 low, and 3 none).

A complete database was built using 25 inputs (Tables S3–S5): 18 ions, 5 vitamins, and 2 PGRs; and 6 outputs (SN, SL, LA, SQ, BC, and H). The use of individual ions and vitamins makes easier the understanding of the specific effects of each avoiding the ion confounding [36,37].

### 4.4. Statistical Analysis

The complete database was firstly analyzed through a traditional statistical comparative analysis using ANOVA (*p* < 0.05) with Tukey’s Studentized Range (HSD) post-hoc test, performed by the software R version 4.1.2 [65].

### 4.5. Artificial Neural Network Analysis

The complete database was analyzed with FormRules^®^ v4.03 [66], which is a neurofuzzy logic software that combines artificial neural networks and fuzzy logic [15,67]. This technology was able to model the database, build “intelligent” mathematical models for each output and express the results as a set of meaningful rules. Modeling was carried out as previously described in detail elsewhere [4,68]. Briefly, this software uses a technology based on the ASMOD algorithm (Adaptive Spline Modelling Of Data) to minimize the number of relevant inputs, reducing the model complexity, and facilitating accuracy with fewer inputs [67,69].

The predictability and accuracy of the neurofuzzy logic model were assessed using the coefficient of determination (Train Set R^2^, Equation (1), and the ANOVA parameters (*f*-ratio) as explained previously [4,68].

(1) $${\mathrm{Train}\;\mathrm{Set}\;R}^{2}=\left(1-\frac{\sum_{i=1}^{n}{\left(y_{i}-y_{i}^{\prime }\right)}^{2}}{\sum_{i=1}^{n}{\left(y_{i}-y_{i}^{\prime \prime }\right)}^{2}}\right)\times 100\;\;\;\;$$

where *y_i_* is the experimental value from the data set, *y_i_*′ is the value calculated by the model, and *y_i_*″ is the mean of the dependent variable. Briefly, for each output, the higher the Train Set R^2^ value, the better the model predictability. R^2^ values higher than 70% indicate reasonable model predictabilities [67]. Additionally, ANOVA evaluates differences between experimental and predicted values. If the ANOVA *f*-ratio is higher than the *f*-critical value there are no statistical significance differences between predicted and experimental values, thus the model is accurate for predictions [68,70].

Several statistical fitness criteria were evaluated to obtain models with the best Train Set R^2^, such as Leave One Out Cross-Validation (LOOCV), Cross-Validation (CV), Bayesian Information Criterion (BIC), Minimum Description Length (MDL), and Structural Risk Minimization (SRM). As described previously [68,71], LOOCV and CV are validation methods that split the data into subgroups that can be used for training and testing. Contrary, BIC, MDL, and SRM are statistical significance methods that use all the data for training. After the evaluation of all of them, it was found that SRM provided the best results, ensuring the highest predictability, accuracy, and easier-to-understand rules. The training parameters selected for modeling are presented in Table 6.

FormRules^®^ software uses a neurofuzzy logic tool to provide the results as ‘IF THEN’ rules, expressed through linguistic tags which go from Low to High. The rules were given a specific membership degree ranging from 0 to 1, making the interpretation easier [18,72].

## 5. Conclusions

The novel strategy of reducing the experimental design space (using DoE) and jointly modeling three independent databases (using ANNs), greatly facilitated the understanding of the results in a simpler way than with the traditional analysis (ANOVA), but also to acquire very useful knowledge about the effect of each media component and their hidden interactions. The ANNs models elucidated the essential role of the mineral nutrients on the growth and quality of micropropagated plants, showing their greater effect compared to vitamins and PGRs. ANNs identified the factors (inputs) that have a special impact on the growth of quality plants and the appearance of physiological disorders, never described previously. Also, ANNs allow narrowing down the range of concentrations to be tested to design a new culture medium by delimiting the space of knowledge (rules) and of design (reducing the number of factors) to be studied. The generated rules easily help to deduce the most suitable ranges of the media components by limiting the ideal ranges of concentration of all the critical factors, to achieve the best plant growth and quality. The next step will be the experimental validation of these results by designing an optimized media using another computer-based tool (based on the combination of ANNs and Genetic Algorithms).

## Figures and Tables

**Table 1:** Neurofuzzy logic model train set R^2^, ANOVA parameters for training (*f*-ratio, degrees of freedom (df1: model and df2: total), *f*-critical value for α = 0.01), and critical factors (inputs selected by the model) for each output (SN: shoot number, SL: shoot length, LA: leaf area, SQ: shoot quality, BC: basal callus, H: hyperhydricity). The inputs with a stronger effect on each output have been highlighted.

Outputs	Submodel	Train Set R^2^ (%)	*f*-Ratio	df1	df2	*f*-Critical (α = 0.01)	Critical Factors
**SN**	**1**	82.3	19.14	17	87	2.18	**Fe^2+^ × Na^+^**
	2						GA_3_
	3						K^+^ × SO_4_^2−^
	4						BAP
**SL**	1	70.3	7.56	20	84	2.10	Na^+−^
	2						Mg^2+^
	3						NO_3_^−^ × K^+^
	4						Vitamin E
	5						BO_3_^−^
	6						GA_3_ × BAP
	**7**						**Co^2+^**
	8						Myo-inositol
**LA**	1	77.7	38.34	7	84	2.86	Na^+^
	2						GA_3_
	**3**						**K^+^ × NO_3_^−^**
	4						SO_4_^2−^
**SQ**	**1**	85.6	49.47	9	84	2.63	**NO_3_^−^**
	2						K^+^
	3						NH_4_^+^
	4						Fe^2+^
	**5**						**MoO_4_^2−^**
	6						BAP
**BC**	**1**	96.0	120.91	14	84	2.30	**PO_4_^3−^ × NH_4_^+^**
	2						SO_4_^2−^
**H**	1	84.4	19.76	18	84	2.16	Co^2+^ × NH_4_^+^
	2						I^−^
	**3**						**SO_4_^2−^ × NO_3_^−^**
	4						Ca^2+^ × Fe^2+^
	5						BAP

**Table 2:** Rules for morpho-physiological growth responses (SN: Shoot number; SL: Shoot length and LA: Leaf area) with their membership degree (MD) generated by neurofuzzy logic. The inputs with the strongest effect indicated by the model have been highlighted.

Rules		[NO_3_^−^]	[K^+^]	[Na^+^]	[SO_4_^2−^]	[Fe^2+^]	[BO_3_^−^]	[Mg^2+^]	Vit E	[Co^2+^]	Myo	BAP	GA_3_		SN	SL	LA	MD
1				Low		Low									High			1.00
**2**				**High**		**Low**									**Low**			**1.00**
**3**				**Low**		**Mid**									**High**			**1.00**
4				High		Mid									Low			1.00
5				Low		High									High			1.00
6				High		High									High			0.79
7													Low		Low			1.00
8													Mid		Low			1.00
9	IF												High	THEN	High			0.58
10			Low		Low										Low			1.00
11			Low		Mid										Low			1.00
12			Low		High										High			1.00
13			Mid		Low										Low			0.75
14			Mid		Mid										High			1.00
15			Mid		High										Low			1.00
16			High		Low										High			1.00
17			High		Mid										Low			1.00
18			High		High										Low			1.00
19												Low			Low			1.00
20												High			Low			0.80
21				Low												High		1.00
22				Mid												High		1.00
23				High												Low		1.00
24								Low								Low		1.00
25								High								High		1.00
26		Low	Low													Low		1.00
27		Low	High													High		1.00
28		High	Low													High		1.00
29		High	High													Low		1.00
30									Low							High		0.94
31	IF								High					THEN		Low		0.91
32							Low									Low		1.00
33							Mid									High		1.00
34							High									High		1.00
35												Low_1	Low			High		1.00
36												Mid_2	Low			Low		1.00
37												Mid_3	Low			Low		1.00
38												High_4	Low			Low		1.00
39												Low_1	High			Low		1.00
40												Mid_2	High			High		1.00
41												Mid_3	High			Low		0.50
42												High_4	High			High		1.00
**43**										**Low**						**High**		**1.00**
44										Mid						Low		1.00
**45**										**High**						**Low**		**1.00**
46											Low					High		0.83
47											High					Low		0.79
**48**				**Low**													**High**	**1.00**
**49**				**High**													**Low**	**1.00**
50													Low				High	0.97
51													High				Low	1.00
52	IF	Low	Low											THEN			Low	1.00
53		High	Low														High	1.00
54		Low	High														Low	0.72
55		High	High														High	0.57
56					Low												Low	1.00
57					High												High	1.00

**Table 3:** Rules for morpho-physiological quality responses (SQ: Shoot quality; BC: basal callus and H: hyperhydricity) with their membership degree (MD) generated by neurofuzzy logic. The inputs with the strongest effect indicated by the model have been highlighted.

Rules		[NO_3_^−^]	[NH_4_^+^]	[K^+^]	[SO_4_^2−^]	[Ca^2+^]	[Co^2+^]	[I^−^]	[Fe^2+^]	[MoO_4_^2−^]	[PO_4_^3−^]	BAP		SQ	BC	H	MD
1									Low					High			1.00
2									High					Low			1.00
3				Low										Low			1.00
4				High										High			1.00
5			Low											Low			1.00
6			High											High			1.00
7	IF									Low			THEN	Low			1.00
8										Mid				High			1.00
9										High				Low			1.00
**10**		**Low**												**High**			**1.00**
11		Mid												High			1.00
**12**		**High**												**Low**			**1.00**
13												Low		High			0.93
14												High		Low			1.00
**15**			**Low**								**Low_1**				**Low**		**1.00**
16			Mid								Low_1				Low		1.00
17			High								Low_1				Low		1.00
18			Low								Mid_2				Low		0.58
19			Mid								Mid_2				Low		1.00
20			High								Mid_2				Low		0.97
**21**			**Low**								**Mid_3**				**High**		**1.00**
22	IF		Mid								Mid_3		THEN		High		1.00
23			High								Mid_3				High		1.00
24			Low								High_4				High		1.00
25			Mid								High_4				High		1.00
26			High								High_4				High		1.00
27					Low										High		1.00
28					Mid										High		0.52
29					High										High		0.78
30								Low								Low	1.00
31								High					THEN			High	1.00
32		Low			Low											Low	1.00
**33**		**High**			**Low**											**Low**	**1.00**
34		Low			Mid											Low	1.00
35		High			Mid											Low	1.00
36		Low			High											High	1.00
**37**		**High**			**High**											**High**	**1.00**
38						Low_1			Low							High	1.00
39	IF					Low_1			High				THEN			High	1.00
40						Mid_2			Low							High	1.00
41						Mid_2			High							High	1.00
42						Mid_3			Low							Low	1.00
43						Mid_3			High							Low	1.00
44						High_4			Low							Low	1.00
45						High_4			High							Low	1.00
46												Low				High	0.75
47												High				Low	1.00
48			Low				Low									High	1.00
49			High				Low									High	1.00
50			Low				High									Low	1.00
51			High				High									Low	1.00

**Table 4:** Ranges (mM and mg L^−1^) and meaning of the ideal levels (Low, Mid, and High) after the fuzzification process by neurofuzzy logic software to achieve the optimal parameter values.

Input	Level	Range
**NH_4_^+^ (mM)**	High	12.37–20.61
**NO_3_^−^ (mM)**	Mid–High	14.35–39.41
**K^+^ (mM)**	Mid	7.28–17.46
**Ca^2+^ (mM)**	Low–Mid_2	0.75–5.89
**Mg^2+^ (mM)**	High	2.44–4.50
**PO_4_^3−^ (mM)**	Mid_3–High_4	1.60–3.75
**SO_4_^2−^ (mM)**	High	2.85–5.20
**Fe^2+^ (mM)**	Low	0.10–0.30
**BO_3_^−^ (mM)**	Mid–High	0.05–0.15
**MoO_4_^2^^−^ (mM)**	Mid	0.0005–0.0012
**Na^+^ (mM)**	Low	0.20–0.60
**Co^2+^ (mM)**	Low	0.00001–0.00008
**I^−^ (mM)**	High	0.0040–0.0075
**Myo (mg L^−1^)**	Low	0–500
**Vit. E (mg L^−1^)**	Low	0.00–0.50
**GA_3_ (mg L^−1^)**	Low	0.00–0.50
**BAP (mg L^−1^)**	Low	0.50–1.50

**Table 5:** Design Expert^®^’s five-factor design for the mineral nutrient and vitamin experiments.

Mineral Nutrient Factors	Media Salts	Range (× MS)
Factor 1	NH_4_NO_3_	0.2–1×
Factor 2	KNO_3_	0.1–1×
Factor 3 (Mesos)	CaCl_2_·2H_2_O	0.25–3×
	MgSO_4_·7H_2_O	
	KH_2_PO_4_	
Factor 4 (Micros)	MnSO_4_·4H_2_O	0.1–1.5×
	ZnSO_4_·7H_2_O	
	H_3_BO_3_	
	KI	
	CuSO_4_·5H_2_O	
	Na_2_MoO_4_·2H_2_O	
	CoCl_2_·6H_2_O	
Factor 5 (Iron)	FeSO_4_·7H_2_O	1–5×
	Na_2_·EDTA	
**Vitamin Factors**	**Vitamins**	**Range (× MS)**
Factor 1	Myo-inositol	0–10×
Factor 2	Thiamine	0–10×
Factor 3	Nicotinic acid	0–10×
Factor 4	Pyridoxine	0–3×
Factor 5	Vitamin E	– ^1^

^1^ Vitamin E concentration levels ranged between 0 and 1.0 mg L^−1^ (see Table S2).

**Table 6:** Train parameters setting for neurofuzzy logic (FormRules^®^ v4.03) software.

FormRules^®^ v4.03
Minimization parameters (ASMOD)
Ridge Regression Factor: 1 × 10^−6^
Model Selection Criteria
Structural Risk Minimization (SRM)
C1_LA, SQ, BC_ = 0.970
C1_SN, H_ = 0.868
C1_SL_ = 0.750
C2 = 4.8
Number of Set Densities: 2
Set Densities: 2, 3
Adapt Nodes: TRUE
Max. Inputs Per SubModel: 2
Max. Nodes Per Input: 15
Minimization parameters (ASMOD)
Ridge Regression Factor: 1 × 10^−6^

## Data Availability

Not applicable.

## Supplementary Materials

The following are available online at https://www.mdpi.com/article/10.3390/plants11101284/s1, Table S1: Ranges (mM and mg L^−1^) and meaning of the levels (Low, Mid and High) after the fuzzification process by neurofuzzy logic software, Table S2: Design Expert^®^’s five-factor design including 33 model points, and MS media as controls, for the mineral nutrient and vitamin experiments; and the 20 combinations of BAP and GA_3_ of the PGR experimental design. Concentrations expressed as × MS, Table S3: Macro and micronutrients (expressed as ion concentrations) of the different culture media based on the five-factor experimental design (0–33) and response values of the parameters (average and standard deviation) used to characterize plant growth. Highest values have been highlighted, Table S4: Vitamin concentration of the different culture media based on the five-factor experimental design (0–33) and response values of the parameters (average and standard deviation) used to characterize plant growth. Highest values have been highlighted, Table S5: PGRs combinations of the different culture media and response values of the parameters (average and standard deviation) used to characterize plant growth. Highest values have been highlighted.
