# Supplementary material for: Artificial Neural Networks Elucidated the Essential Role of Mineral Nutrients versus Vitamins and Plant Growth Regulators in Achieving Healthy Micropropagated Plants
Source: Plants (Basel). 2022 May 11;11(10):1284. doi: 10.3390/plants11101284 (PMC9146087; doi:10.3390/plants11101284)
Supplement: Supplementary file 1 [file plants-11-01284-s001.zip › Supplementary.pdf]

Table S1. Ranges (mM and mg L^{-1}) and meaning of the levels (Low, Mid, and High) after the fuzzification process by neurofuzzy logic software.

	2 levels			3 levels				4 levels				
	**Low**		**High**	**Low**	**Mid**		**High**	**Low_1**	**Mid_2**		**Mid_3**	**High_4**
NH_4^+ (mM)	4.12	12.37	20.61	4.12	8.25	16.49	20.61					
NO_3^- (mM)	6.00	22.71	39.41	6.00	14.35	31.06	39.41					
K^+ (mM)	2.19	12.37	22.55	2.19	7.28	17.46	22.55					
Ca^{2+} (mM)								0.75	2.81	5.89	7.95	8.98
Mg^{2+} (mM)	0.38	2.44	4.50									
PO_4^{3-} (mM)								0.31	0.74	1.60	2.89	3.75
SO_4^{2-} (mM)	0.49	2.85	5.20	0.49	1.67	4.02	5.20					
Fe^{2+}/EDTA (mM)	0.10	0.30	0.50	0.10	0.20	0.40	0.50					
BO_3^- (mM)				0.01	0.05	0.12	0.15					
MoO_4^{2-} (mM)				0.0001	0.0005	0.0012	0.0015					
Na^+ (mM)	0.20	0.60	1.00	0.20	0.40	0.80	1.00					
Co^{2+} (mM)	0.00001	0.00008	0.00016	0.00001	0.00005	0.00012	0.00016					
I^- (mM)	0.0005	0.0040	0.0075									
Myo (mg L^{-1})	0.0	500.0	1000.0									
Vit. E (mg L^{-1})	0.0	0.5	1.0									
GA_3 (mg L^{-1})	0.00	0.50	1.00	0.00	0.25	0.75	1.00					
BAP (mg L^{-1})	0.50	1.50	2.50					0.50	1.00	1.75	2.25	2.50

Table S2. Design Expert®'s five-factor design including 33 model points, and MS media as controls, for the mineral nutrient and vitamin experiments; and the 20 combinations of BAP and GA_3 of the PGR experimental design. Concentrations expressed as × MS.

**1**	**2**	**Factor 1**	**Factor 2**	**Factor 3**	**Factor 4**	**Factor 5**
**Experimental design**	**Treatments**	**NH_4NO_3**	**KNO_3**	**Mesos**	**Micros**	**Iron**
	1	0.51	0.66	1.65	1.50	3.04
	2	1.00	0.66	2.59	0.10	1.00
	3	1.00	1.00	2.37	1.50	1.08
	4	0.20	0.24	3.00	0.97	1.84
	5	0.90	0.10	0.25	0.10	1.60
	6	0.20	1.00	0.94	0.10	5.00
	7	0.20	1.00	0.94	0.10	5.00
	8	0.40	1.00	0.25	1.50	1.00
	9	0.81	0.10	0.25	1.40	5.00
	10	1.00	1.00	3.00	0.39	5.00
	11	0.20	0.33	3.00	0.10	5.00
	12	0.81	0.10	0.25	1.40	5.00
**Mineral**	13	0.80	0.10	2.99	1.29	1.00

**nutrients**	14	0.40	0.10	0.25	0.10	5.00
	15	0.20	0.10	2.67	1.50	4.20
	16	1.00	0.33	0.25	1.50	1.00
	17	0.61	0.64	1.90	0.14	3.54
	18	0.20	0.76	0.25	0.10	1.00
	19	0.30	1.00	3.00	1.22	5.00
	20	1.00	0.10	2.38	0.10	4.46
	21	1.00	1.00	0.90	1.50	4.78
	22	0.20	0.76	0.25	1.50	5.00
	23	0.20	0.76	0.25	1.50	5.00
	24	1.00	1.00	0.25	0.10	3.30
	25	1.00	0.35	1.52	0.94	3.10
	26	0.20	0.76	3.00	1.50	1.00
	27	0.24	1.00	3.00	0.10	2.00
	28	0.24	1.00	3.00	0.10	2.00
	29	0.20	0.76	3.00	1.50	1.00
	30	0.32	0.10	2.34	0.10	1.00
	31	0.72	0.72	0.90	0.69	1.00
	32	0.20	0.10	0.80	1.36	1.00
	33	1.00	0.34	3.00	1.50	5.00
	MS	1.00	1.00	1.00	1.00	1.00
		**Myo-inositol**	**Thiamine**	**Nicotinic acid**	**Pyridoxine**	**Vitamin E (mg L^{-1})[1]**
	1	0.00	4.00	0.00	0.00	0.00
	2	0.00	8.50	0.00	3.00	1.00
	3	0.00	0.55	8.76	3.00	1.00
	4	10	10	10	0.00	0.00
	5	10	10	0.00	1.78	1.00
	6	0.00	7.40	10	0.00	0.66
	7	10	0.00	6.00	3.00	0.00
	8	0.50	0.00	0.00	3.00	0.26
	9	5.95	0.00	10	0.00	1.00
	10	0.00	7.40	10	0.00	0.66
	11	10	10	0.00	1.78	1.00
	12	0.00	10	4.76	1.72	0.03
	13	10	7.15	0.00	1.96	0.00
	14	10	3.05	10	1.78	0.60
**Vitamins**	15	10	3.05	10	1.78	0.60
	16	8.75	0.55	0.00	3.00	1.00
	17	3.80	6.95	10	3.00	0.00
	18	10	10	5.00	3.00	0.55
	19	5.25	3.30	5.30	0.32	0.11

	20	4.75	4.35	4.76	1.54	0.52
	21	10	10	5.00	3.00	0.55
	22	4.75	4.35	4.76	1.54	0.52
	23	0.00	10	4.30	1.06	1.00
	24	4.75	10	0.00	0.16	0.57
	25	1.82	1.70	1.82	2.86	0.95
	26	10	0.00	0.00	0.00	0.38
	27	10	7.00	3.86	0.00	1.00
	28	0.00	6.05	0.00	1.92	0.38
	29	0.00	0.00	0.56	0.82	1.00
	30	0.00	0.00	10	1.08	0.00
	31	5.15	10	10	1.68	1.00
	32	5.96	6.55	0.00	3.00	0.62
	33	4.75	4.35	4.76	1.54	0.52
	MS	1.00	1.00	1.00	1.00	–
		BAP	GA_3	–	–	–
	1	0.00	0.00	–	–	–
	2	0.50	0.00	–	–	–
	3	1.00	0.00	–	–	–
	4	1.50	0.00	–	–	–
	5	2.00	0.00	–	–	–
	6	2.50	0.00	–	–	–
	7	0.50	0.10	–	–	–
	8	1.00	0.10	–	–	–
	9	1.50	0.10	–	–	–
**PGRs**	10	2.00	0.10	–	–	–
	11	2.50	0.10	–	–	–
	12	0.50	0.50	–	–	–
	13	1.00	0.50	–	–	–
	14	1.50	0.50	–	–	–
	15	2.00	0.50	–	–	–
	16	2.50	0.50	–	–	–
	17 (MS)	1.00	1.00	–	–	–
	18	1.50	1.00	–	–	–
	19	2.00	1.00	–	–	–
	20	2.50	1.00	–	–	–

[1] Vitamin E concentration levels are expressed as mg L^{-1}, as the MS medium does not contain this organic compound.

Table S3. Macro and micronutrients (expressed as ion concentrations) of the different culture media based on the five-factor experimental design (0-33) and response values of the parameters (average and standard deviation) used to characterize plant growth. Highest values have been highlighted.

Media	NH_4^+	NO_3^-	K^+	Ca^{2+}	Mg^{2+}	PO_4^{3-}	SO_4^{-2}	Cl^-	Fe^{2+}/EDTA	BO_3^-	Mn^{2+}	Zn^{2+}	Cu^{2+}	MoO_2^{2-}	Na^+	Co^{2+}	I^-	SN	SL	LA	SQ	BC	H
1	10.47	22.84	14.44	4.95	2.48	2.06	2.98	9.89	0.30	0.15	0.15	0.045	0.00015	0.0015	0.61	0.00016	0.0075	2.7 ± 0.9	1.8 ± 0.5	20.6 ± 6.3	3.8 ± 0.7	4.0 ± 0.0	2.9 ± 0.5
2	20.61	33.06	15.68	7.74	3.88	3.23	4.00	15.49	0.10	0.01	0.01	0.003	0.00001	0.0001	0.20	0.00001	0.0005	2.4 ± 1.4	1.9 ± 0.5	**40.7 ± 10.9**	3.2 ± 1.2	4.0 ± 0.0	1.8 ± 0.9
3	20.61	39.41	21.76	7.09	3.55	2.96	3.86	14.17	0.11	0.15	0.15	0.045	0.00015	0.0015	0.22	0.00016	0.0075	1.1 ± 0.7	1.4 ± 0.3	26.1 ± 12.8	4.4 ± 0.7	4.0 ± 0.0	2.8 ± 0.5
4	4.21	8.62	8.17	8.98	4.50	3.75	4.81	17.96	0.18	0.10	0.10	0.029	0.00010	0.0010	0.37	0.00010	0.0048	3.3 ± 1.5	1.7 ± 0.4	12.0 ± 4.0	3.5 ± 0.5	4.0 ± 0.0	1.8 ± 1.0
5	18.55	20.43	2.19	0.75	0.38	0.31	0.55	1.50	0.16	0.01	0.01	0.003	0.00001	0.0001	0.32	0.00001	0.0005	2.9 ± 1.2	1.4 ± 0.2	7.1 ± 3.4	3.1 ± 0.5	4.0 ± 0.0	3.0 ± 0.0
6	4.12	22.92	19.96	2.81	1.41	1.17	1.92	5.61	0.50	0.01	0.01	0.003	0.00001	0.0001	1.00	0.00001	0.0005	0.6 ± 0.9	1.4 ± 0.4	6.8 ± 3.2	2.5 ± 0.9	4.0 ± 0.0	3.0 ± 0.0
7	4.12	22.92	19.96	2.81	1.41	1.17	1.92	5.61	0.50	0.01	0.01	0.003	0.00001	0.0001	1.00	0.00001	0.0005	0.3 ± 0.8	1.3 ± 0.4	7.8 ± 4.4	2.2 ± 0.9	4.0 ± 0.0	3.0 ± 0.0
8	8.25	27.04	19.11	0.75	0.38	0.31	0.67	1.50	0.10	0.15	0.15	0.045	0.00015	0.0015	0.20	0.00016	0.0075	**3.4 ± 1.6**	1.3 ± 0.2	8.7 ± 3.9	2.8 ± 0.5	3.5 ± 0.5	2.9 ± 0.3
9	16.73	18.61	2.20	0.75	0.38	0.31	1.06	1.50	0.50	0.14	0.14	0.042	0.00014	0.0014	1.00	0.00015	0.0070	0.3 ± 0.6	1.1 ± 0.1	3.1 ± 1.7	1.7 ± 0.5	4.0 ± 0.0	3.0 ± 0.0
10	20.61	39.41	22.54	8.98	4.50	3.75	5.05	17.96	0.50	0.04	0.04	0.012	0.00004	0.0004	1.00	0.00004	0.0019	0.3 ± 0.5	1.3 ± 0.4	9.2 ± 3.6	2.3 ± 1.0	4.0 ± 0.0	2.2 ± 1.0
11	4.21	10.31	9.86	8.98	4.50	3.75	5.02	17.96	0.50	0.01	0.01	0.003	0.00001	0.0001	1.00	0.00001	0.0005	1.0 ± 0.9	1.3 ± 0.2	6.0 ± 2.1	1.7 ± 0.7	4.0 ± 0.0	1.9 ± 1.0
12	16.73	18.61	2.20	0.75	0.38	0.31	1.06	1.50	0.50	0.14	0.14	0.042	0.00014	0.0014	1.00	0.00015	0.0070	0.4 ± 0.7	1.2 ± 0.2	3.8 ± 1.2	1.5 ± 0.5	3.1 ± 0.2	3.0 ± 0.0
13	16.57	18.45	5.62	8.94	4.48	3.73	4.75	17.88	0.10	0.13	0.13	0.039	0.00013	0.0013	0.20	0.00014	0.0064	2.6 ± 1.0	1.7 ± 0.4	17.0 ± 5.5	3.6 ± 0.7	4.0 ± 0.0	1.9 ± 0.7
14	8.16	10.04	2.19	0.75	0.38	0.31	0.89	1.50	0.50	0.01	0.01	0.003	0.00001	0.0001	1.00	0.00001	0.0005	0.1 ± 0.3					
15	4.12	6.00	5.22	7.99	4.01	3.34	4.62	15.98	0.42	0.15	0.15	0.045	0.00015	0.0015	0.84	0.00016	0.0075	0.3 ± 0.5	1.2 ± 0.0	4.1 ± 1.8	1.3 ± 0.6	4.0 ± 0.0	2.9 ± 0.3
16	20.61	26.89	6.60	0.75	0.38	0.31	0.67	1.50	0.10	0.15	0.15	0.045	0.00015	0.0015	0.20	0.00016	0.0075	2.9 ± 1.4	1.8 ± 0.4	14.0 ± 3.4	3.1 ± 0.6	3.9 ± 0.2	2.6 ± 0.7
17	12.66	24.76	14.47	5.69	2.85	2.37	3.23	11.37	0.35	0.01	0.01	0.004	0.00001	0.0001	0.71	0.00001	0.0007	0.3 ± 0.6	1.3 ± 0.2	8.7 ± 2.3	2.3 ± 0.9	4.0 ± 0.0	3.0 ± 0.0
18	4.12	18.35	14.54	0.75	0.38	0.31	0.49	1.50	0.10	0.01	0.01	0.003	0.00001	0.0001	0.20	0.00001	0.0005	2.7 ± 0.8	1.6 ± 0.3	8.8 ± 2.8	2.9 ± 0.5	2.0 ± 0.0	2.9 ± 0.3
19	6.26	25.05	22.55	8.98	4.50	3.75	5.16	17.96	0.50	0.12	0.12	0.036	0.00012	0.0013	1.00	0.00013	0.0061	0.8 ± 0.9	1.6 ± 0.4	14.5 ± 6.4	3.4 ± 1.0	4.0 ± 0.0	2.7 ± 0.8
20	20.61	22.49	4.85	7.13	3.57	2.97	4.03	14.25	0.45	0.01	0.01	0.003	0.00001	0.0001	0.89	0.00001	0.0005	0.2 ± 0.4	1.5 ± 0.3	6.1 ± 3.7	2.1 ± 0.7	4.0 ± 0.0	2.9 ± 0.3
21	20.61	39.41	19.92	2.68	1.35	1.12	2.02	5.37	0.48	0.15	0.150	0.045	0.00015	0.0015	0.96	0.00016	0.0075	0.0 ± 0.0					
22	4.12	18.35	14.55	0.75	0.38	0.31	1.07	1.50	0.50	0.15	0.15	0.045	0.00015	0.0015	1.00	0.00016	0.0075	1.4 ± 0.7	1.2 ± 0.2	4.1 ± 1.4	1.7 ± 0.5	1.0 ± 0.0	3.0 ± 0.0
23	4.12	18.35	14.55	0.75	0.38	0.31	1.07	1.50	0.50	0.15	0.15	0.045	0.00015	0.0015	1.00	0.00016	0.0075	0.9 ± 1.0	1.1 ± 0.1	4.0 ± 1.6	1.7 ± 0.5	1.2 ± 0.7	3.0 ± 0.0
24	20.61	39.41	19.11	0.75	0.38	0.31	0.72	1.50	0.33	0.01	0.01	0.003	0.00001	0.0001	0.66	0.00001	0.0005	1.2 ± 0.9	1.5 ± 0.4	4.7 ± 2.0	1.9 ± 0.8	4.0 ± 0.0	2.6 ± 0.9
25	20.61	27.14	8.43	4.53	2.27	1.89	2.71	9.07	0.31	0.09	0.09	0.028	0.00009	0.0010	0.62	0.00010	0.0047	1.9 ± 0.6	1.8 ± 0.4	16.2 ± 5.3	3.7 ± 0.7	4.0 ± 0.0	2.9 ± 0.5
26	4.12	18.43	18.07	8.98	4.50	3.75	4.80	17.96	0.10	0.15	0.15	0.045	0.00015	0.0015	0.20	0.00016	0.0075	0.7 ± 0.8	1.9 ± 0.9	21.7 ± 10.3	4.2 ± 0.8	3.6 ± 0.5	2.8 ± 0.4
27	4.95	23.74	22.54	8.98	4.50	3.75	4.72	17.96	0.20	0.01	0.01	0.003	0.00001	0.0001	0.40	0.00001	0.0005	2.1 ± 1.2	1.7 ± 0.4	28.5 ± 8.5	3.9 ± 0.9	4.0 ± 0.0	1.4 ± 0.7
28	4.95	23.74	22.54	8.98	4.50	3.75	4.72	17.96	0.20	0.01	0.01	0.003	0.00001	0.0001	0.40	0.00001	0.0005	1.8 ± 1.1	2.0 ± 0.6	29.9 ± 13.8	4.1 ± 0.8	4.0 ± 0.0	1.8 ± 0.7
29	4.12	18.43	18.07	8.98	4.50	3.75	4.80	17.96	0.10	0.15	0.15	0.045	0.00015	0.0015	0.20	0.00016	0.0075	1.0 ± 1.0	1.8 ± 0.6	18.9 ± 9.3	3.5 ± 1.2	3.7 ± 0.5	2.4 ± 0.8
30	6.51	8.39	4.80	7.00	3.51	2.92	3.63	14.01	0.10	0.01	0.01	0.003	0.00001	0.0001	0.20	0.00001	0.0005	2.1 ± 1.4	1.5 ± 0.3	9.3 ± 3.9	1.4 ± 0.5	4.0 ± 0.0	1.0 ± 0.0
31	14.92	28.46	14.67	2.68	1.35	1.12	1.54	5.36	0.10	0.07	0.07	0.021	0.00007	0.0007	0.20	0.00007	0.0034	2.1 ± 1.0	**2.1 ± 0.7**	28.3 ± 9.3	**4.7 ± 0.5**	4.0 ± 0.0	2.7 ± 0.5
32	4.12	6.00	2.89	2.39	1.20	1.00	1.48	4.79	0.10	0.14	0.14	0.041	0.00014	0.0014	0.20	0.00014	0.0068	1.3 ± 1.1	1.3 ± 0.3	5.7 ± 2.9	2.4 ± 0.8	4.0 ± 0.0	2.9 ± 0.3
33	20.61	26.98	10.12	8.98	4.50	3.75	5.20	17.96	0.50	0.15	0.15	0.045	0.00015	0.0015	1.00	0.00016	0.0075	0.6 ± 1.0	1.6 ± 0.4	10.4 ± 5.5	2.6 ± 0.8	4.0 ± 0.0	2.4 ± 0.9
MS	20.61	39.41	20.05	2.99	1.50	1.25	1.73	5.99	0.10	0.10	0.10	0.030	0.00010	0.0010	0.20	0.00011	0.0050	1.9 ± 1.1	1.6 ± 0.3	28.7 ± 9.3	4.1 ± 0.4	4.0 ± 0.0	2.6 ± 0.6

Table S4. Vitamin concentration of the different culture media based on the five-factor experimental design (0-33) and response values of the parameters (average and standard deviation) used to characterize plant growth. Highest values have been highlighted.

Media	Myo	Thia	Nic	Pyr	VitE	Gly	SN	SL	LA	SQ	BC	H
1	0	4.00	0.00	0.00	0.00	2.0	1.7 ± 0.7	1.4 ± 0.2	20.5 ± 6.2	3.8 ± 0.7	4.0 ± 0.0	2.9 ± 0.5
2	0	8.50	0.00	1.50	1.00	2.0	2.3 ± 0.7	1.6 ± 0.3	25.5 ± 5.2	3.9 ± 0.4	4.0 ± 0.0	2.9 ± 0.4
3	0	0.55	4.38	1.50	1.00	2.0	**2.4 ± 1.5**	1.5 ± 0.4	26.3 ± 6.9	3.9 ± 0.3	4.0 ± 0.0	2.6 ± 0.6
4	1000	10.00	5.00	0.00	0.00	2.0	1.4 ± 0.8	1.4 ± 0.2	19.5 ± 7.7	3.7 ± 0.6	4.0 ± 0.0	2.8 ± 0.6
5	1000	10.00	0.00	0.89	1.00	2.0	2.1 ± 1.2	**1.8 ± 0.4**	**27.7 ± 5.7**	4.3 ± 0.5	4.0 ± 0.0	2.8 ± 0.4
6	0	7.40	5.00	0.00	0.66	2.0	2.1 ± 1.2	1.7 ± 0.3	23.1 ± 7.5	4.3 ± 0.8	4.0 ± 0.0	2.6 ± 0.7
7	1000	0.00	3.00	1.50	0.00	2.0	1.9 ± 1.1	1.5 ± 0.3	17.2 ± 5.9	3.7 ± 0.6	4.0 ± 0.0	2.7 ± 0.7
8	50	0.00	0.00	1.50	0.26	2.0	1.8 ± 0.7	1.7 ± 0.4	21.8 ± 4.8	4.3 ± 0.5	4.0 ± 0.0	2.9 ± 0.3
9	595	0.00	5.00	0.00	1.00	2.0	1.9 ± 0.7	1.4 ± 0.3	19.6 ± 3.7	4.0 ± 0.8	4.0 ± 0.0	2.7 ± 0.7
10	0	7.40	5.00	0.00	0.66	2.0	2.1 ± 0.9	1.6 ± 0.4	26.2 ± 6.5	4.0 ± 0.9	4.0 ± 0.0	2.7 ± 0.7
11	1000	10.00	0.00	0.89	1.00	2.0	1.9 ± 1.1	1.4 ± 0.2	24.3 ± 6.6	4.3 ± 0.6	4.0 ± 0.0	2.8 ± 0.4
12	0	10.00	2.38	0.86	0.03	2.0	1.5 ± 0.9	1.6 ± 0.2	18.8 ± 6.7	3.6 ± 0.8	4.0 ± 0.0	2.5 ± 0.8
13	1000	7.15	0.00	0.98	0.00	2.0	2.2 ± 1.0	1.6 ± 0.4	27.3 ± 12	4.0 ± 0.8	4.0 ± 0.0	2.5 ± 0.8
14	1000	3.05	5.00	0.89	0.60	2.0	2.0 ± 0.8	1.6 ± 0.4	26.1 ± 5.4	4.5 ± 0.6	4.0 ± 0.0	2.8 ± 0.6
15	1000	3.05	5.00	0.89	0.60	2.0	2.1 ± 1.0	1.6 ± 0.4	22.1 ± 5.5	4.2 ± 0.4	4.0 ± 0.0	2.9 ± 0.5
16	875	0.55	0.00	1.50	1.00	2.0	2.1 ± 1.0	1.4 ± 0.3	26.3 ± 6.4	4.1 ± 0.6	4.0 ± 0.0	2.7 ± 0.6
17	380	6.95	5.00	1.50	0.00	2.0	1.6 ± 0.7	1.6 ± 0.4	20.5 ± 4.8	4.1 ± 0.5	4.0 ± 0.0	2.7 ± 0.7
18	1000	10.00	2.50	1.50	0.55	2.0	2.0 ± 1.1	1.5 ± 0.3	24.4 ± 7.1	4.1 ± 1.1	4.0 ± 0.0	2.6 ± 0.8
19	525	3.30	2.65	0.16	0.11	2.0	1.9 ± 0.8	1.6 ± 0.4	26.7 ± 6.5	4.2 ± 0.8	4.0 ± 0.0	2.5 ± 0.8
20	475	4.35	2.38	0.77	0.52	2.0	2.2 ± 0.9	1.4 ± 0.3	26.1 ± 3.8	4.5 ± 0.5	4.0 ± 0.0	2.9 ± 0.4
21	1000	10.00	2.50	1.50	0.55	2.0	2.0 ± 1.0	1.3 ± 0.2	25.4 ± 7.8	4.2 ± 0.6	4.0 ± 0.0	2.9 ± 0.3
22	475	4.35	2.38	0.77	0.52	2.0	1.8 ± 0.9	1.4 ± 0.2	24.9 ± 7.1	4.5 ± 0.5	4.0 ± 0.0	2.9 ± 0.3
23	0	10.00	2.15	0.53	1.00	2.0	1.5 ± 1.0	1.3 ± 0.2	15.9 ± 5.4	3.8 ± 0.7	4.0 ± 0.0	3.0 ± 0.0
24	475	10.00	0.00	0.08	0.57	2.0	1.8 ± 1.3	1.6 ± 0.5	16.6 ± 7.2	3.9 ± 1.1	4.0 ± 0.0	2.7 ± 0.7
25	182	1.70	0.91	1.43	0.95	2.0	1.7 ± 1.2	1.6 ± 0.3	15.4 ± 7.1	3.9 ± 1.0	4.0 ± 0.0	2.9 ± 0.5
26	1000	0.00	0.00	0.00	0.38	2.0	1.3 ± 0.9	1.4 ± 0.4	14.2 ± 6.0	3.7 ± 1.0	4.0 ± 0.0	2.7 ± 0.7
27	1000	7.00	1.93	0.00	1.00	2.0	1.2 ± 1.1	1.4 ± 0.2	14.8 ± 6.3	3.8 ± 0.8	4.0 ± 0.0	2.9 ± 0.4
28	0	6.05	0.00	0.96	0.38	2.0	2.2 ± 1.2	1.6 ± 0.3	20.7 ± 8.2	4.3 ± 0.9	4.0 ± 0.0	3.0 ± 0.0
29	0	0.00	0.28	0.41	1.00	2.0	1.9 ± 1.2	1.4 ± 0.2	21.4 ± 6.0	4.4 ± 0.5	4.0 ± 0.0	3.0 ± 0.0
30	0	0.00	5.00	0.54	0.00	2.0	1.5 ± 1.0	1.6 ± 0.3	22.1 ± 7.5	4.5 ± 0.8	4.0 ± 0.0	2.9 ± 0.3
31	515	10.00	5.00	0.84	1.00	2.0	1.7 ± 0.9	1.4 ± 0.3	22.0 ± 5.2	4.5 ± 0.5	4.0 ± 0.0	3.0 ± 0.0
32	596	6.55	0.00	1.50	0.62	2.0	1.4 ± 0.9	1.3 ± 0.3	20.9 ± 6.2	4.0 ± 0.5	4.0 ± 0.0	2.9 ± 0.3
33	475	4.35	2.38	0.77	0.52	2.0	1.7 ± 1.0	1.5 ± 0.4	25.3 ± 10.7	4.0 ± 0.5	4.0 ± 0.0	2.7 ± 0.7
MS	100	0.10	0.50	0.50	0.00	2.0	1.8 ± 1.0	1.6 ± 0.4	22.1 ± 8.0	4.1 ± 0.7	4.0 ± 0.0	2.9 ± 0.4

Table S5. PGRs combinations of the different culture media and response values of the parameters (average and standard deviation) used to characterize plant growth. Highest values have been highlighted.

Media	GA3	BAP	SN	SL	LA	SQ	BC	H
H1	0.00	0.00	0.0 ± 0.0					
H2	0.00	0.50	0.8 ± 0.8	1.5 ± 0.6	32.4 ± 13.1	3.9 ± 0.8	4.0 ± 0.0	2.8 ± 0.6
H3	0.00	1.00	0.8 ± 1.1	1.6 ± 0.3	32.6 ± 16.3	3.3 ± 1.0	4.0 ± 0.0	2.7 ± 0.7
H4	0.00	1.50	1.8 ± 1.0	1.6 ± 0.9	33.6 ± 12.0	3.8 ± 0.6	4.0 ± 0.0	2.9 ± 0.3
H5	0.00	2.00	1.3 ± 0.5	1.3 ± 0.3	**34.7 ± 7.0**	4.0 ± 0.7	4.0 ± 0.0	2.8 ± 0.6
H6	0.00	2.50	1.3 ± 1.1	1.5 ± 0.3	28.8 ± 10.1	3.5 ± 0.7	4.0 ± 0.0	2.5 ± 0.8
H7	0.10	0.50	1.3 ± 1.1	2.1 ± 0.9	31.5 ± 7.1	3.9 ± 0.5	4.0 ± 0.0	2.7 ± 0.8
H8	0.10	1.00	0.9 ± 1.3	1.4 ± 0.3	31.5 ± 8.0	3.8 ± 0.4	4.0 ± 0.0	2.9 ± 0.3
H9	0.10	1.50	0.9 ± 1.1	1.4 ± 0.1	27.2 ± 9.3	3.3 ± 0.7	4.0 ± 0.0	2.7 ± 0.7
H10	0.10	2.00	1.5 ± 1.1	1.6 ± 0.4	30.3 ± 9.1	3.8 ± 0.6	4.0 ± 0.0	2.5 ± 0.8
H11	0.10	2.50	1.1 ± 1.1	1.2 ± 0.3	24.3 ± 6.6	3.4 ± 0.7	4.0 ± 0.0	2.3 ± 1.0
H12	0.50	0.50	0.7 ± 0.9	1.5 ± 0.3	30.3 ± 9.7	3.9 ± 0.9	4.0 ± 0.0	2.7 ± 0.7
H13	0.50	1.00	1.0 ± 1.0	1.7 ± 0.3	26.5 ± 10.7	3.5 ± 1.2	4.0 ± 0.0	2.5 ± 0.9
H14	0.50	1.50	1.2 ± 1.0	1.8 ± 0.5	27.0 ± 5.3	**4.3 ± 0.6**	4.0 ± 0.0	2.8 ± 0.6
H15	0.50	2.00	0.8 ± 0.9	1.4 ± 0.1	28.9 ± 4.3	3.9 ± 0.3	4.0 ± 0.0	3.0 ± 0.0
H16	0.50	2.50	1.3 ± 0.9	1.7 ± 0.4	25.3 ± 5.1	3.7 ± 0.9	4.0 ± 0.0	2.5 ± 0.9
H17 (MS)	1.00	1.00	1.5 ± 1.2	2.0 ± 0.6	24.0 ± 5.1	3.8 ± 0.8	4.0 ± 0.0	2.8 ± 0.6
H18	1.00	1.50	1.8 ± 1.4	**2.3 ± 0.7**	19.6 ± 3.7	3.4 ± 1.1	4.0 ± 0.0	2.4 ± 0.9
H19	1.00	2.00	1.5 ± 1.5	1.7 ± 0.3	22.9 ± 7.0	3.7 ± 0.6	4.0 ± 0.0	2.6 ± 0.7
H20	1.00	2.50	**2.7 ± 2.4**	1.8 ± 0.5	21.3 ± 7.2	3.3 ± 0.8	4.0 ± 0.0	2.4 ± 0.9
